# Academic coaching as a pedagogy to facilitate the navigation of complexity across the health professions education continuum

**DOI:** 10.3389/fmed.2025.1523741

**Published:** 2025-06-06

**Authors:** Svetlana M. King, Ricardo Carnicer Hijazo, Shafeena Anas, Naomi Low-Beer

**Affiliations:** ^1^Prideaux Discipline of Clinical Education, Flinders Health and Medical Research Institute, College of Medicine and Public Health, Flinders Health and Medical Research Institute, College of Medicine and Public Health, Prideaux Discipline of Clinical Education, Flinders University, Adelaide, SA, Australia; ^2^Brunel Medical School, Brunel University of London, London, United Kingdom

**Keywords:** academic coaching, self-regulated learning, reflective practice, pedagogy, learner support, health professions education

## Abstract

Maintaining currency and managing complexity in a rapidly evolving healthcare environment requires health professionals to be competent in monitoring and regulating their own learning. While health professional educators can scaffold learners to develop this competency, maintaining these skills in the absence of ongoing, structured support can prove challenging. Academic coaching is a pedagogical approach that supports learners to develop as self-regulated learners. This longitudinal support can facilitate learners’ capacity to plan, monitor and evaluate their performance and apply these skills to novel contexts, which is needed to navigate the increasingly complex healthcare environment. In this paper, we introduce the intersecting concepts of self-regulated learning and academic coaching. We suggest ways that academic coaching can be used to support learners in the health professions to continually improve their practice and develop their capacity to cope with complexity. We draw on our experiences of implementing academic coaching into two medical programs in the UK and Australia (school-leaver and graduate entry programs, respectively) and offer considerations for implementing academic coaching across the health professions education continuum.

## Introduction

Healthcare professionals are working in an era where uncertainty has become the norm, accelerated by behavioral factors and recent worldwide events including the COVID-19 pandemic, civil unrest, and climate change. While each of these events is significant and impacts health and healthcare, challenges become increasingly complex when circumstances overlap. Consider a patient with COVID-19 and underlying respiratory conditions brought about by poor air quality, obesity, and smoking. This patient’s condition could be exacerbated by mental health issues triggered by forced migration. This example serves to highlight the reality of changing patient profiles.

Internationally, multimorbidity is on the rise, with aging populations increasingly presenting with multiple lifelong conditions (1). For example, the Australian Institute of Health and Welfare (2) reported that in 2022, 15.4 million Australians (61%) were living with a long-term health condition and 9.7 million (38%) had two or more lifelong conditions. In England, Head and colleagues (3) found an increase in prevalence of basic morbidity (two or more chronic conditions) and complex multimorbidity (at least three chronic conditions impacting three or more body systems) from a random sample of general practice records (*n* = 991,243) between 2004 (30.8% basic; 15.1% complex) to 2019 (52.8% basic; 32.7% complex).

Patients with multimorbidity require complex management, typically involving frequent interactions with healthcare providers and a greater emphasis on patient-centered care (4). Clinicians need to develop bespoke management approaches that rely on multiprofessional collaboration to facilitate complex problem-solving. Additionally, the rise of international migration due to civil unrest and climate change means that clinicians need to adapt their communication to provide culturally appropriate care for increasingly diverse communities. Alongside these challenges, healthcare professionals must remain up to date with technological advances (e.g., generative artificial intelligence) that can enhance diagnostics, patient care, and data management while recognizing the ethical dimensions of their use. This begs the question—how do we prepare and support health professionals to manage this complexity?

In terms of preparation, university-based medical education programs typically adopt a traditional “block” approach to curriculum, where students learn patterns of disease within disciplinary boundaries and/or body systems. While this approach has utility in terms of the scaffolding it affords, it does not reflect changing patient profiles. Consequently, this places responsibility on students to navigate complex cases as they begin interacting with patients in the community. Yet, effectively managing patients with multimorbidity is not solely a challenge facing students; rather, it persists in clinical practice.

Clinical management is inhibited by fragmented healthcare system policies and structures that support single condition care and specialization, with clinicians’ decision-making informed by limited supportive evidence and clinical uncertainty (5). This can create a training-practice chasm, where clinical reasoning is tested, requiring extrapolation and expert use of technology-driven decision support systems to manage complexity. Bridging this gap requires clinicians to develop adaptive expertise (6) and maintain their knowledge and skills throughout their careers (7), including the management and use of infinite information. The ability to evaluate, derive meaning and learn from clinical encounters forms part of the standards set by regulatory bodies around the world including the UK (8), Australia and New Zealand (9). To address this requirement, clinicians must become experts in their own learning—they must become master adaptive learners (10).

## Adaptive and self-regulated learning

Managing and solving complex clinical problems requires agility and adaptive expertise. In turn, this requires skills to identify and address gaps in current knowledge to improve practice (10).

Self-regulated learning is a process whereby learners actively set learning goals and develop action plans which involve monitoring and regulating their motivation, cognition, behaviors (11), and emotions (12). It focuses on learning controlled by the learner, rather than a covert reaction to teaching (13). Self-regulated learners have metacognitive awareness (13); recognizing strengths and weaknesses, and proactively addressing areas for development (14). Using wide-ranging evidence, including feedback, self-regulated learners evaluate their abilities to develop goals and actionable plans. Progress is monitored by evaluating strategies and making adjustments as needed (13). Self-regulated learning is associated with academic achievement and mental health (15) with a role in mitigating the risk of burnout (16).

Self-assessment, goal setting, and monitoring require self-reflection (10, 17) and reflective practice—a process of engaging in critical thinking about one’s own professional activities to analyze decisions and reasoning (18), aligned with principles of reflective learning (19). Reflective practice is a mechanism whereby learners navigate complex situations (20) to derive meaning from their experiences through critical analysis (21), and analyze feedback for improvement. In reflective practice, the learner analyzes their experiences, often from multiple perspectives, to identify key learning (and action) points. Hence, reflective practice is fundamental to self-regulated learning and among the core requirements for safe, effective clinical practice. For example, the Medical Board of Australia’s (22) code of conduct includes the expectation that medical practitioners will demonstrate self-awareness and regularly engage in self-reflection. Similarly, the UK General Medical Council expects students to use reflective practice and self-directed learning for continuous learning and improvement (8). Murdoch-Eaton and Sandars (23) extend this idea, suggesting that reflection lies at the heart of professional practice.

Various models, introduced in the 1980s following on from John Dewey’s (24) work, continue to be used to facilitate reflective practice [e.g., Kolb’s (25) experiential learning cycle, Gibbs’ (26) reflective model, and Schon’s (27) reflective practice model]. What these models have in common is the link between reflection, metacognition, and action. Learners can engage in reflection *for* action to identify learning needs and develop goals and plans to address these needs. They can reflect *in* action to evaluate the effectiveness of learning strategies and monitor progress to achieve their goals. Finally, learners can reflect *on* action, adopting a retrospective stance to evaluate achievement of their goals.

It is clear from the literature and from professional standards that adaptive and self-regulated learning are fundamental competencies required of medical practitioners. Indeed, the capacity to identify and address gaps in knowledge and skills is critical to navigating the complexities of clinical practice. So, how can we support health professional students and clinicians to develop and maintain these competencies throughout their careers?

## The need for learning support

Considered essential for safe clinical practice (15), self-regulated learning is increasingly becoming an accreditation requirement for medical education programs. While programs exist to facilitate self-regulated learning [e.g., the Self-Directed Learning in Medical Education model (28)], there is likely to be variability in medical graduates’ preparedness for self-regulated learning as they commence clinical practice. Furthermore, junior doctors are likely to work alongside experienced practitioners with little or no training in self-regulated learning, despite requirements for self-reflection and goal setting as part of annual competency review processes. Additionally, the workplace culture may not actively and explicitly facilitate the development of self-regulated learning and adaptive expertise. Given the increasing complexities of clinical practice coupled with the emotional elements of self-regulated learning, facilitating adaptive expertise is critical to effective ongoing learning in the workplace (10) and clinician wellbeing.

It could be argued that medical courses inadvertently select for students who are effective learners; evidenced by sustained academic achievement required to be competitive for admission. Yet, crowded medical education curricula can challenge even the brightest of learners, with an identified link between burnout and perceptions of the learning environment (29). Once enrolled, students may be prompted to re-evaluate their existing, previously highly effective learning strategies in favor of those offering increased efficiency. Additionally, the shift from memorizing information to pass assessments, to understanding and applying knowledge to real-world clinical practice scenarios inherently requires different study techniques. While learners may come with expertise in passing assessments, they do not necessarily have the skills to monitor and evaluate their learning, as Zheng et al. (30). Hence, we argue that self-regulated learning characteristics are not necessarily innate. Just as health professional courses support students to develop foundational knowledge and skills upon which to continue to build throughout their career, they must prioritize explicit teaching and support to develop as self-regulated learners.

Self-regulated learning is emphasized as a core competency of the medical programs at Flinders University (Australia) and Brunel University of London (UK). In both programs, medical students maintain a reflective practice portfolio wherein they reflect on their learning experiences, evaluate their strengths and areas for development, set learning goals, and monitor progress in achieving these goals. This portfolio is maintained for the duration of enrolment, providing students with opportunities to evaluate their progress longitudinally. This forms a key component of programmatic assessment—a longitudinal, integrated learning and assessment system in which progression decisions are made based on multiple datapoints over time (31). Here, learners use assessment results and feedback to evaluate their progress over time, moving from a focus on assessment *of* learning, to increasingly prioritize assessment *for* learning (32).

While programmatic assessment and the portfolio are structurally integrated into our programs, their presence alone is limited in facilitating self-regulated learning. Learners can find it difficult to transfer metacognitive processes across contexts, particularly in and around key transition points. For example, we have observed that students find it challenging to apply self-regulated learning principles in transitioning from the structured, predictable university learning environment to the unstructured, unpredictable clinical setting. Similarly, learners can encounter the challenge of transfer as they transition to medical student, to postgraduate trainee/resident and, later, to independent specialist practitioner. Successfully navigating these transitions and distinct learning contexts, while attending to health and wellbeing, requires scaffolding and support. The same, we argue, applies in clinical practice. A study of specialist trainee reflections found substantial variability in the level of portfolio engagement, suggesting the need for structured support (33). Without ongoing support, there is a risk that metacognitive and reflective practice processes will become de-emphasized and deprioritized, reducing clinicians’ capacity to develop adaptive expertise.

## Academic coaching

Coaching is a learner-centered pedagogy (34) that focuses on continuous improvement (35). The concept of coaching in medical education has been adapted from other disciplines such as business, music and sport (36). It is based on the premise that medicine is a similarly elite pursuit, characterized by continual refinement. While there are similarities with mentoring (37), coaching is distinct with its targeted, improvement focus (38) and goal orientation (39). The coaching relationship is typically finite (40) and learner-driven (39, 41), while mentoring is often longer-term and centered around the mentor’s expertise (42).

Coaching aligns with the principles of reflective (19) and adult learning (43) theory, introducing a collaborative, dialog-based learning partnership. It can take different forms including performance-based coaching, involving direct observation in a clinical setting (44), and academic coaching. In this paper, we focus on academic coaching, defining it as “… an interactive, longitudinal, relational, learner-centered process” (45) that supports learners to develop as self-regulated learners and reflective practitioners (46, 47).

Academic coaching differs from performance-based coaching which routinely involves direct observation and real-time feedback to enhance technical proficiency for improved patient care (48). Richardson et al. make a similar distinction in their Competence By Design model, referring to performance-based coaching as “coaching in the moment” and academic coaching as “coaching over time” (49).

Academic coaching is becoming integral in medical education programs that adopt programmatic assessment (50) and competency-based medical education (35). It focuses on reviewing learners’ accounts of experiences, feedback analysis and self-assessment (50–52) to support metacognition and reflective practice (41) and facilitate transfer across novel contexts (53). Academic coaches do this by actively assisting learners to: derive meaning from their experiences; challenge their assumptions (34); evaluate their abilities using evidence; set learning goals (54, 55); and develop action plans (41, 53). Because academic coaching is learner-centered (50), coaches provide tailored guidance (40, 56), working with learners to create bespoke, targeted improvement plans (56, 57). This may involve specific performance-based coaching provided by an expert in the identified area for improvement.

Academic coaches require strong communication skills, the capacity to make informed observations and judgments (58), the ability to identify and recognize learning patterns by reframing information (34, 40), and the ability to ask powerful questions (59) and motivate and encourage the learner (60) to maintain personal accountability (41). Academic coaching involves scaffolding (46) as requirements evolve over time in response to the developing learner-coach relationship, and the learner’s changing needs as they enhance their capacity for independent self-regulation (41).

Academic coaches play a crucial role in supporting learners to recognize the multiple domains that contribute to, and impact on their learning. For example, coaching can support learners to evaluate their motivations to learn for gateway postgraduate examinations. Coaching can facilitate early identification of learners who are struggling to meet expectations (61) and promote the adoption of adaptive help-seeking behaviors (42, 51) by connecting them with relevant support services. Coaches can support learners to develop effective time management strategies tailored to unique workplace challenges alongside personal circumstances and ameliorate the risk of burnout (62). Academic coaching can also facilitate preparedness for leadership roles, and foster communication skills such as advocacy and negotiation. Additionally, academic coaching has been linked to enhanced academic performance and program completion (53, 63).

Academic coaching offers health services a mechanism to support clinicians across the continuum to continue to develop adaptive expertise to meet increasingly complex healthcare demands. Indeed, several coaching programs have been developed that focus on facilitating the transition to clinical practice [e.g., (64, 65)]. Offering such support can equip clinicians with the skills to expertly handle uncertainty, manage their own health and wellbeing, and maintain their commitment to lifelong learning. This could enhance career satisfaction, facilitate motivation and accountability to follow through with learning plans, and support preparation for increasing levels of responsibility along the learning continuum. Ultimately, ongoing support for self-regulated learning through academic coaching could improve patient care and outcomes.

In addition to learner benefits, academic coaches themselves have much to gain from the process including increased job satisfaction and the development of new skills (60). For example, a Flinders University project (underway) has revealed that academic coaches experience parallel development as self-regulated learners through their involvement with the program. One participant, an experienced consultant clinician, noted that he has become a better practitioner because he actively and routinely seeks feedback from various sources to improve his practice. Additionally, we support academic coaches to recognize the transferability of their coaching skillset to other contexts such as clinical supervision, postgraduate education, and trainee development. Hence, the academic coach role affords the opportunity for experienced clinicians to develop their own capacity for self-regulated learning through the support they provide to junior colleagues. This would foster a strong improvement-focused workplace culture that promotes the development of adaptive expertise among every clinician, ultimately optimizing patient care.

## Considerations for embedding academic coaching in practice

Our academic coaching experiences are situated in the university context and we have previously published recommendations for program-level design and implementation of academic coaching (45). Our intention, therefore, is not to replicate these recommendations but, rather, adopt a broader perspective to present individual-, organizational- and system-level considerations for embedding academic coaching in clinical contexts.

At the individual level:

1. *Foster learner-coach relationships based on mutual trust and respect*

Academic coaching is founded on an effective relationship between the learner and the coach (53, 66). Indeed, the quality of the relationship is fundamental to coaching success (51, 58). This is critically important because learners need confidence to be vulnerable in disclosing uncertainties and weaknesses. This is particularly challenging within the culture of medicine which privileges competence and success over development and failure (35). Productive coaching relationships can also facilitate adaptive and proactive help-seeking (51). The learner may seek out their coach to debrief (positive and negative) learning experiences which may not otherwise be discussed with colleagues. While debriefing has therapeutic benefits, in a coaching relationship, it also has an educative focus. Where a therapeutic relationship is warranted, the academic coach can facilitate help-seeking by encouraging access to counseling. This can mitigate the risk of burnout—a well-recognized issue among medical practitioners—and foster wellbeing, creating a healthier workforce.

2. *Support learners to bridge the theory-practice gap*

Encourage learners to seek opportunities to consolidate their learning using multiple strategies, fostering metacognition, critical thinking, and reflection. For example, if a learner identifies the need to refine their technique for a particular procedural skill, use open questions to promote exploration of multiple perspectives: Which aspects of the procedure do you perform well, and how do you know you perform these steps well? Which aspects of the procedure would you like to improve, and how do you know these aspects require improvement? (*informed self-assessment*, *reflection*) What resources will enhance your understanding of the procedure, and how do you know these resources are evidence-informed? (*critical thinking*) How will you know whether the resources you have selected are helping to address the gap you have identified? (*reflection*) How will you address the aspects of the procedure you have identified as requiring improvement? How and when will you know you have improved? (*metacognition*).

At an organizational level:

3. *Embed academic coaching into health services*

Recognizing and embedding academic coaching as a professional activity in the workplace can facilitate clinician engagement with the program. Having such programs staffed by health service employees can foster a culture of continuous, contextualized learning in the workplace. Creating opportunities for clinicians to contribute as academic coaches can also enhance career satisfaction (34) and their own self-regulated learning practices, as seen in the Flinders program.

4. *Provide opportunities for academic coaches to develop self-regulated learning and coaching skills*

Like other programs (47, 67), establishing a community of practice among academic coaches has been critical in our academic coaching programs. While this builds individual and collective coaching capacity, it also creates space for reflective practice. Encouraging academic coaches to seek feedback from learners and fellow coaches fosters a continuous improvement mindset for all those involved in academic coaching. Additionally, it facilitates transfer of self-regulation and coaching practices to other contexts where coaches provide pedagogical support (e.g., clinical supervision).

At a system level:

5. *Integrate academic coaching across the health professions education learning continuum*

Academic coaching has the potential to enhance more meaningful clinician engagement with regular performance reviews and continuing professional development. Here, the coach could support the learner to evaluate their learning needs, and partner with the learner to deliberately and purposefully select professional development activities that address the learner’s needs while simultaneously addressing professional regulation requirements. This could facilitate discussion and review of holistic issues that affect performance and wellbeing, extending the focus of annual competency reviews beyond maintaining skills. In this way, academic coaching would move from a ‘nice-to-have’ initiative to become a critical vehicle to foster ongoing self-regulated learning within the continuing professional development context.

## Conclusion

Clinicians require skills in self-regulated learning to continue to adapt to the evolving healthcare landscape. Academic coaching can foster these skills by guiding learners through the processes of self-assessment, goal setting, and reflective practice. Through targeted support and feedback, academic coaches can assist clinicians to identify knowledge and skills gaps and develop effective strategies for continuous learning and improvement. This powerful pedagogical practice not only enhances clinical reasoning and decision-making but also empowers clinicians to navigate complexity and uncertainty with confidence throughout their careers, ultimately improving patient care.

## Data Availability

The original contributions presented in the paper are included in the article. Further inquiries can be directed to the corresponding author.
